# Novel biological aqua crust enhances *in situ* metal(loid) bioremediation driven by phototrophic/diazotrophic biofilm

**DOI:** 10.1186/s40168-023-01549-3

**Published:** 2023-05-18

**Authors:** Guobao Wang, Xiuran Yin, Zekai Feng, Chiyu Chen, Daijie Chen, Bo Wu, Chong Liu, Jean Louis Morel, Yuanyuan Jiang, Hang Yu, Huan He, Yuanqing Chao, Yetao Tang, Rongliang Qiu, Shizhong Wang

**Affiliations:** 1 School of Environmental Science and Engineering, Sun Yat-Sen University, Guangzhou, 510006 China; 2 Guangdong Provincial Key Laboratory of Environmental Pollution Control and Remediation Technology, Sun Yat-Sen University, Guangzhou, 510275 China; 3 Microbial Ecophysiology Group, University of Bremen, Bremen, Germany; 4 Institute of Agricultural Resources and Environment, Guangdong Academy of Agricultural Sciences, Guangzhou, 510640 China; 5 Laboratoire Sols Et Environnement, UMR 1120, Université de Lorraine, INRAE, 54518 Vandoeuvre-Lès-Nancy, France; 6 Guangdong Laboratory for Lingnan Modern Agriculture, Guangdong Provincial Key Laboratory of Agricultural & Rural Pollution Abatement and Environmental Safety, College of Natural Resources and Environment, South China Agricultural University, Guangzhou, 510642 China

**Keywords:** Biological aqua crust, Metal(loid) bioremediation, Metagenomics, Biofilm formation

## Abstract

**Background:**

Understanding the ecological and environmental functions of phototrophic biofilms in the biological crust is crucial for improving metal(loid) (*e.g.* Cd, As) bioremediation in mining ecosystems. In this study, in combination with metal(loid) monitoring and metagenomic analysis, we systematically evaluated the effect of biofilm in a novel biological aqua crust (biogenic aqua crust—BAC) on *in situ* metal(loid) bioremediation of a representative Pb/Zn tailing pond.

**Results:**

We observed strong accumulation of potentially bioavailable metal(loid)s and visible phototrophic biofilms in the BAC. Furthermore, dominating taxa *Leptolyngbyaceae* (10.2–10.4%, *Cyanobacteria*) and *Cytophagales* (12.3–22.1%, *Bacteroidota*) were enriched in biofilm. Along with predominant heterotrophs (*e.g. Cytophagales* sp.) as well as diazotrophs (*e.g. Hyphomonadaceae* sp.), autotrophs/diazotrophs (*e.g. Leptolyngbyaceae* sp.) in phototrophic biofilm enriched the genes encoding extracellular peptidase (*e.g.* family S9, S1), CAZymes (*e.g.* CBM50, GT2) and biofilm formation (*e.g. OmpR*, *CRP* and *LuxS*), thus enhancing the capacity of nutrient accumulation and metal(loid) bioremediation in BAC system.

**Conclusions:**

Our study demonstrated that a phototrophic/diazotrophic biofilm constitutes the structured communities containing specific autotrophs (*e.g. Leptolyngbyaceae* sp.) and heterotrophs (*e.g. Cytophagales* sp.), which effectively control metal(loid) and nutrient input using solar energy in aquatic environments. Elucidation of the mechanisms of biofilm formation coupled with metal(loid) immobilization in BAC expands the fundamental understanding of the geochemical fate of metal(loid)s, which may be harnessed to enhance *in situ* metal(loid) bioremediation in the aquatic ecosystem of the mining area.

Video Abstract

**Supplementary Information:**

The online version contains supplementary material available at 10.1186/s40168-023-01549-3.

## Background

Biological aqua crust (biogenic aqua crust—BAC) is a complex organo-mineral system composed of biotic components including microorganisms and their secretions, and abiotic components such as mineral particles in aquatic ecosystems [1]. Similar to the biological soil crust (BSC) that is known as an ecosystem engineer of ecological succession in arid and semi-arid areas [2–4], the BAC is expected to be capable of not only enriching large numbers of specific autotrophs and heterotrophs but also regulating the local and global biogeochemical fluxes of carbon and nitrogen even in extreme environments such as metal(loid)-rich (*e.g.* Cd, As) water ecosystems [1, 5, 6]. Unlike the BSC in previous studies [7, 8], the BAC investigated in this study showed the characteristics of a sponge-like porous structure and the functions of metal(loid) immobilization in aquatic habitats. The BAC has a potential to improve the quality of metal(loid)-contaminated water bodies and has, therefore, been considered a suitable candidate for investigations of microbial interactions with minerals and metal(loid)s derived from mining activities [1, 9, 10]. However, our understanding of metal(loid) bioavailability and microbial communities in BAC of polymetallic water bodies remains limited.

Microorganisms especially autotrophic cyanobacteria often secret large amounts of extracellular polymeric substances (EPS), create microenvironments and form biofilms to survive in metal(loid)-rich habitats such as mine drainage [11]. As excellent primary producers, photoautotrophic microorganisms control microbial structure and strongly influence nutrient (*e*.*g*. carbon and nitrogen) cycles in the BSC of extreme environments (*e.g.* desert areas) [6]. Owing to their autotrophic properties, photoautotrophic microorganisms (*e.g.* filamentous cyanobacteria) are likely to occupy unique nutritional feeding niches in the form of biofilms in oligotrophic streams [12]. Therefore, biofilms are recognized as potential and sustainable candidates for the remediation of oligotrophic and metal(loid)-contaminated environments [13]. Moreover, mineral particles such as clay minerals (*e.g.* kaolinite) and mine waste (*e.g.* goethite), which are widely distributed in BAC [1], are important drivers of bacterial (*e.g. Bacillus subtilis*) biofilm establishment and formation [14, 15]. Thus, biofilms are likely integral components of BAC microstructures. However, the roles of microbiota in biofilm formation and nutrient (*e*.*g*. carbon and nitrogen) fixation in the BAC of aquatic ecosystems have not been described, and further research is required to elucidate the metabolic functions of microbes in the BAC of polymetallic and oligotrophic waters.

The combination of metagenomics and microscopy could provide in-depth insights into how phototrophic biofilms within BAC control metal(loid) bioremediation in oligotrophic waters. This study aimed to: 1) characterize the metal(loid) bioavailability and biofilm morphology in the BAC, 2) describe the microbial populations and major microbiota in BAC, and 3) predict the metabolic potentials of the predominant microbiota related to biofilm formation, nutrition cycles, and metal(loid) bioremediation in BAC. The findings of this study provide insights into the taxonomic composition and metabolic potential of the innate microbiota of BAC in both nutrient deficiency and metal(loid)-rich water bodies.

## Results

### Metal(loid) immobilization and biofilm formation

**Fig. 1:**
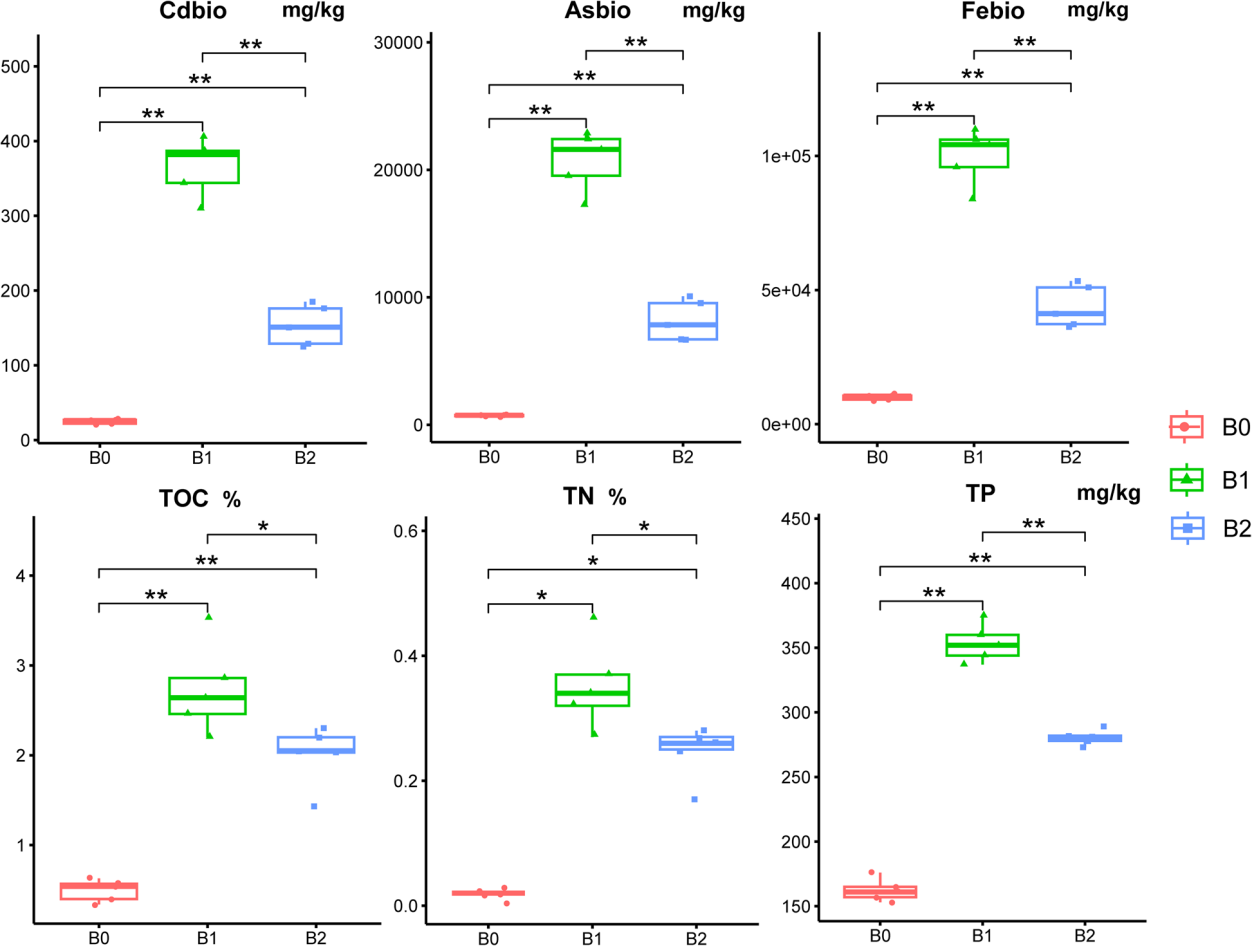
Concentrations of potential bioavailable metal(loid)s (*i.e.* Cd_bio_, As_bio_, Fe_bio_) and nutrients (*i.e.* TOC, TN, TP) in studied samples (*n* = 5). Notably, the TOC data were cited from our previous study [1]. B_0_ represents the source tailings, and B_1-2_ stand for the biological aqua crusts collected at Sites 1 and 2. Kruskal–Wallis test was performed to multiply compare the statistical significance. The symbol of “*” and “**” stand for the significant variation at the levels of 0.05 and 0.01, respectively. The “ns” represents no significant difference

In an aquatic ecosystem, the potentially bioavailable portion of pollutants represents an important fraction that can be either incorporated by organisms or mobilized to the downstream ecosystem. Thus, the evaluation of the bioavailable fraction of metal(loid)s (*e.g.* Cd_bio_, As_bio_, and Fe_bio_) would contribute to a direct understanding of the metal(loid) release potential related to microorganisms in the biological aqua crust (BAC). As shown in Fig. 1, the concentrations of potentially bioavailable metal(loid)s such as Cd_bio_ (24.8 mg kg^−1^) and As_bio_ (0.72 g kg^−1^), were relatively high in source tailings (B_0_). Interestingly, the concentrations of potentially bioavailable metal(loid)s in BAC (B_1-2_) were even significantly (*P* < 0.05) higher than those in B_0_ by 6.2–14.8 times for Cd_bio_, 4.4–10.0 times for Fe_bio_, and 11.4–28.8 times for As_bio_, which is consistent with our previous results of Cd, As and Fe concentrations [1]. The concentrations of Cd_bio_, As_bio_, and Fe_bio_ significantly decreased from upstream B_1_ to downstream B_2_. In addition, major biogenic elements, such as carbon, nitrogen, and phosphorus, were studied to directly reflect microbial activity and nutrient status. The concentrations of total organic carbon (TOC, 0.5%) [1], total nitrogen (TN, 0.02%), and total phosphorus (TP, 162 mg kg^−1^) were extremely low in B_0_. In addition to TOC (4.0–5.4 times) [1], the BAC (B_1-2_) showed 13.7–19.6 times higher TN, and 1.7–2.2 times higher TP than B_0_. The concentrations of the TN and TP also significantly decreased from B_1_ to B_2_.

**Fig. 2:**
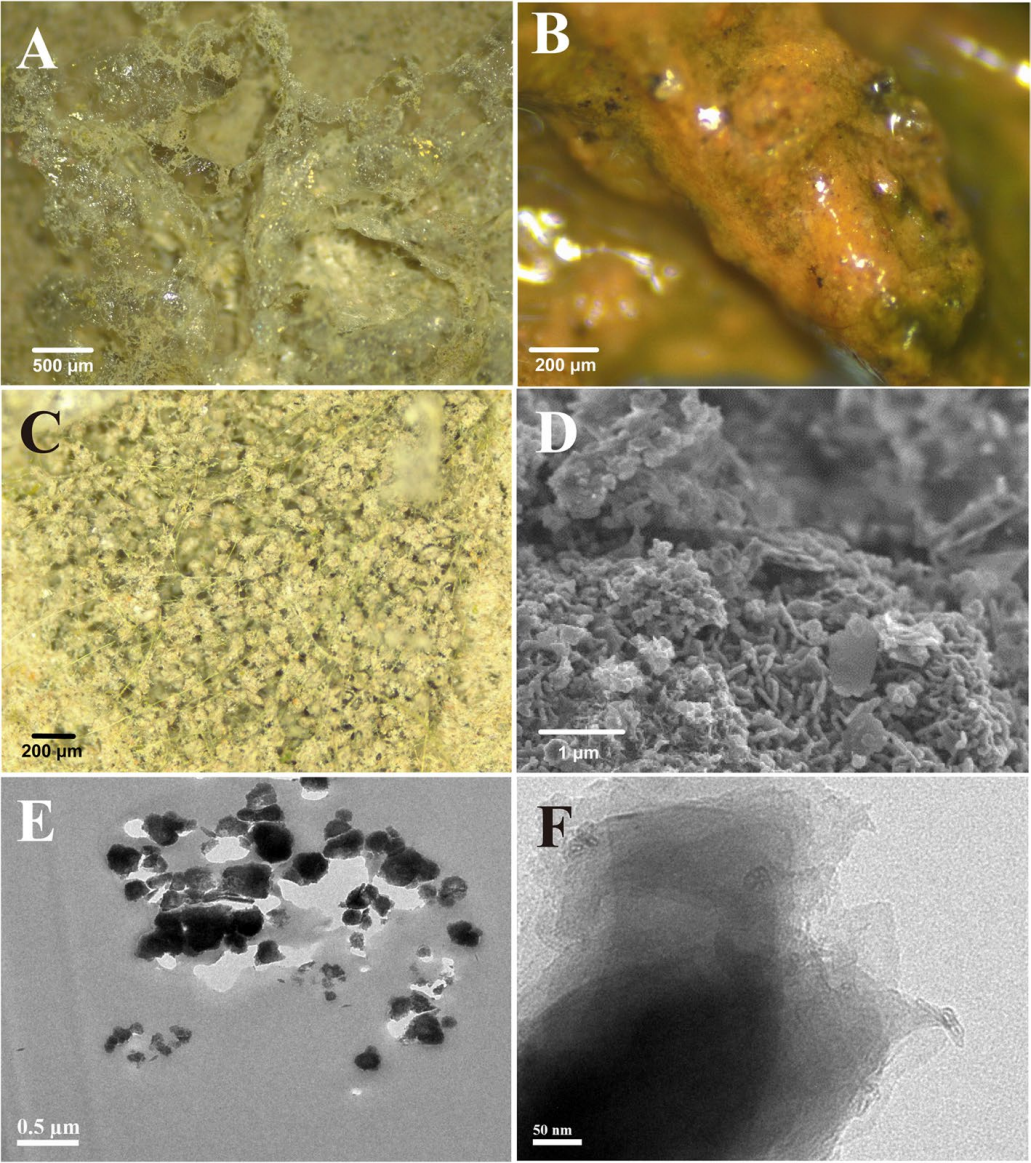
Surface morphology and microstructure of the biological aqua crust (BAC). **A** the freeze-dried EPS and minerals revealed by stereomicroscope; (**B**) organo-mineral aggregates visualized by stereomicroscope in original conditions; (**C**) aggregates including filamentous organisms and mineral particles revealed by stereomicroscope; (**D**) microorganisms and minerals revealed by SEM; (**E**, **F**) EPS and mineral particles revealed by TEM in the BAC. The photos of morphology and microstructure were originated from upstream BAC, and the layout was conducted using Adobe Illustrator CC (v23.0) software

The morphology and microstructure of BAC in aquatic ecosystems were further visualized using advanced microscopies (*i.e.* stereomicroscope, SEM, and TEM) to provide a direct insight into the potential aggregated forms of EPS, biofilm, and minerals (Fig. 2). In the freeze-dried condition, many transparent and membranous EPS-like- and biofilm-like structures cemented with mineral particles and filamentous microorganisms were observed in BAC (Fig. 2A). In the original wet conditions, viscous aggregates containing potential EPS, microorganisms, and mineral particles [1] were also observed in the BAC (Fig. 2B). Notably, these EPS and biofilm could act like a “mineral glue” [16] which therefore adhere and bind the mineral particles to form the organo-mineral aggregates in BAC of aquatic systems. Moreover, the inner microstructure visualized by stereomicroscopy and SEM showed that filamentous microorganisms could strongly interact and bind with mineral particles in the form of organo-mineral aggregates (Fig. 2C-D). The organo-mineral aggregates comprising EPS [17] and mineral particles were further identified by TEM (Fig. 2E-F), which suggested that microbial metabolic activities related to biofilm formation might be critical for the formation of aggregates and the accumulation of metal(loid)s and biogenic elements (*e.g.* carbon and nitrogen).

### Microbial composition and genome phylogeny

**Fig. 3:**
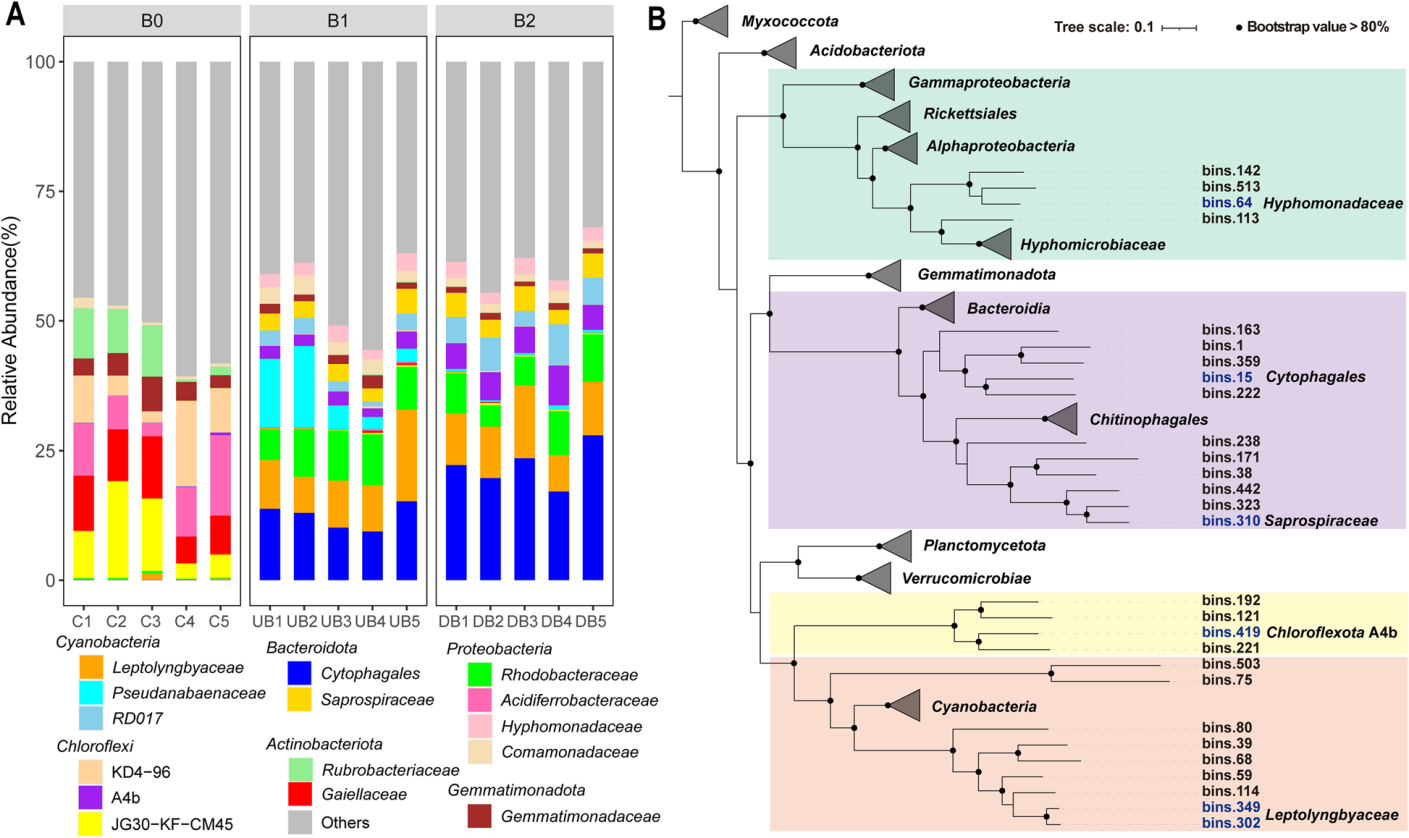
Microbial composition at the family level (**A**) and maximum-likelihood phylogeny of metagenome-assembled genomes (MAGs, **B**) in studied samples. A total of 15 families collectively occupied 54.7% of total average abundance were visualized in bar plot. B_0_ (C1–C5) represents the source tailings, B_1_ (UB1–UB5) and B_2_ (DB1–DB5) stand for the BACs collected at Sites 1 and 2, respectively. Phylum names in phylogeny were associated with the colors, *i.e.* light green: *Proteobacteria*; purple: *Bacteroidota*; light yellow: *Chloroflexi*; brown: *Cyanobacteria*

16S rRNA gene amplicon sequencing and metagenomic binning were used to reveal the structure of the microbial community and the phylogenetic features of some taxa abundant in BAC (Fig. 3). A total of 46 phyla were identified based on classifiable sequences. Interestingly, microbiota in the BAC were dominated by *Cyanobacteria* (20.3–24.7% of total sequences), *Bacteroidota* (21.0–31.2%) and *Proteobacteria* (26.3–32.0%) with top 3 high abundance, while *Actinobacteriota* (27.4%), *Chloroflexi* (23.1%) and *Proteobacteria* (22.2%) stood for the most dominant lineages in the source tailings (Fig. S1). At the family level, *Leptolyngbyaceae* (10.2–10.4% with the mean relative abundance, *Cyanobacteria*) and *Cytophagales* (12.3–22.1%, *Bacteroidota*) were the top 2 most dominant taxa, followed by the taxa of *Rhodobacteraceae* (7.0–8.5%, *Proteobacteria*), *Pseudanabaenaceae* (0.5–7.7%, *Cyanobacteria*), *Chloroflexi* A4b (2.5–5.5%), *Cyanobacteria* RD017 (2.4–5.5%), *Saprospiraceae* (3.4–4.1%, *Bacteroidota*) and *Hyphomonadaceae* (2.6%, *Proteobacteria*) in the BAC (Fig. 3A, Table S1). However, the *Chloroflexi* JG30-KF-CM45 (9.8%), *Gaiellaceae* (*Actinobacteriota*, 9.1%) and *Acidiferrobacteraceae* (*Proteobacteria*, 8.8%) were the three most abundant families in the source tailings.

In addition, a total of 109 near-complete MAGs (completeness ≥ 90%, potential contamination ≤ 5%) were obtained to further reveal the genomic features of high-abundant taxa at the strain level in the BAC (Fig. 3B, Table S3). All bin genomes retrieved from the nine phyla were used for phylogenetic analyses, and *Proteobacteria* (30 MAGs), *Bacteroidota* (19 MAGs), *Cyanobacteria* (12 MAGs), and *Planctomycetota* (11 MAGs) were the top four dominant phyla. In accordance with the 16S rRNA gene amplicon findings, bins.15 with the greatest sequencing read depth was retrieved as *Cytophagales* (Fig. 3B, Table S3). The MAGs of both bins.302 and bins.349 were annotated as the family *Leptolyngbyaceae*, while bins.64, bins.310 and bins.419 were affiliated with the families *Hyphomonadaceae*, *Saprospiraceae* and *Chloroflexi* A4b, respectively. As the core microbiota in BAC, MAGs may affect biofilm formation.

### Key genes related to biofilm formation in MAGs

**Fig. 4:**
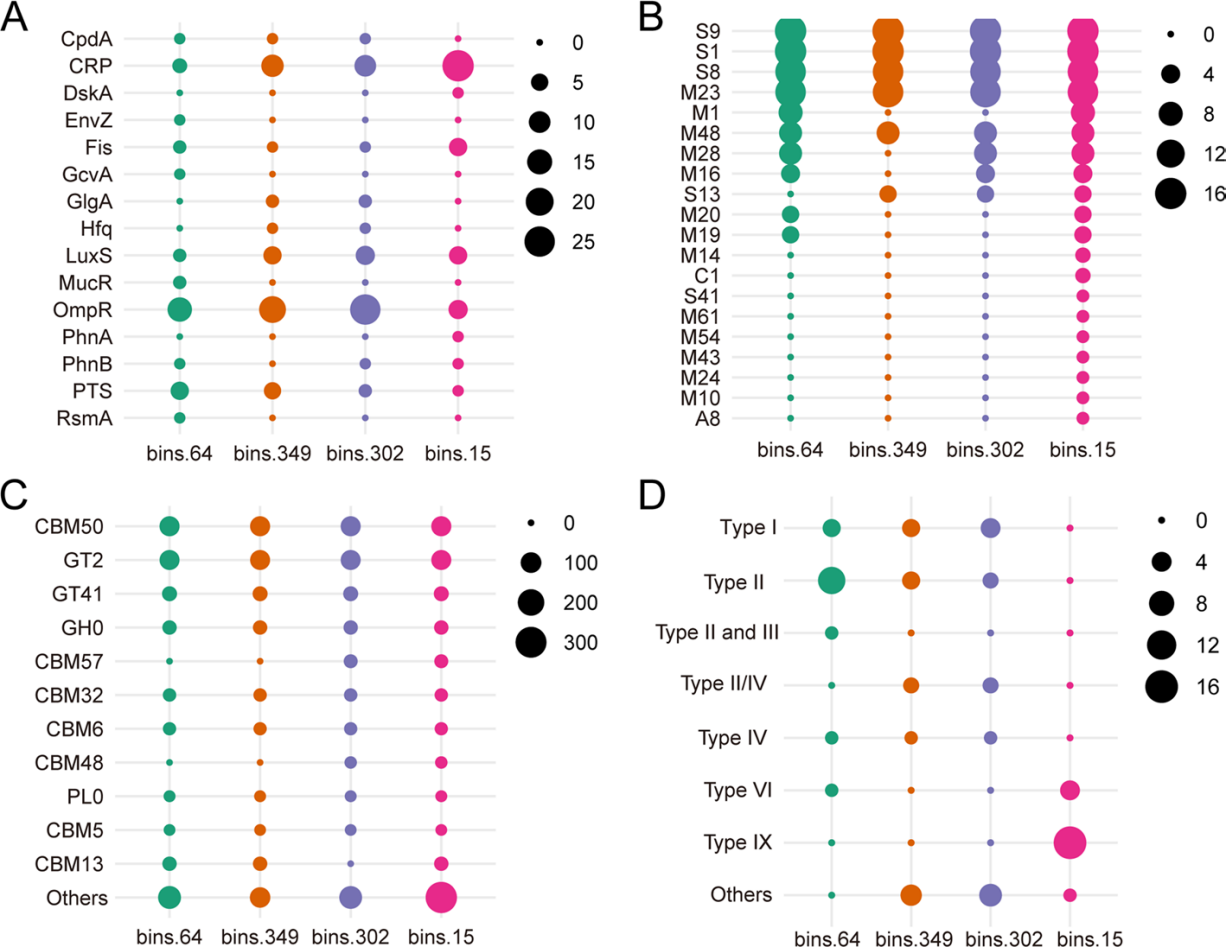
The genes encoding biofilm formation (**A**), extracellular peptidase (**B**), CAZymes (**C**) and secretion type (**D**) in selected MAGs of biological aqua crust. Only high abundance genes related to CAZyme secretion were selected for visualization

The phototrophic *Leptolyngbyaceae* sp. (*i.e.* bins.349, bins.302), heterotrophic *Hyphomonadaceae* sp. (*i.e.* bins.64) and *Cytophagales* sp. (*i.e.* bins.15) abundant in BAC were further selected to study the functional genes encoding biofilm formation, extracellular peptidase, carbohydrate-active enzymes (CAZymes), and secretion type (Fig. 4, Table S5-8). Based on the metagenomic analysis, all four MAGs showed strong metabolic potential for biofilm formation. In detail, *OmpR* was the most dominant gene encoding biofilm formation in *Leptolyngbyaceae* sp. (*i.e.* bins.349, bins.302) and *Hyphomonadaceae* sp. (*i.e.* bins.64), whereas *CRP* (Cyclic AMP receptor protein) was the most dominant gene in *Cytophagales* sp. (*i.e.* bins.15) (Fig. 4A). Notably, *LuxS* is abundant in both *Leptolyngbyaceae* sp. and *Cytophagales* sp. In all the above MAGs, families S9, S1, S8 and M23 were the top 4 high-abundant gene sets related to extracellular peptidase secretion (Fig. 4B), whereas families CBM50 and GT2 were the dominant genes encoding secretory CAZymes (Fig. 4C). Interestingly, *Cytophagales* sp. (*i.e.* bins.15) showed the strongest potential for secretion of both extracellular peptidases and CAZymes. Moreover, type I, type II, and type IV secretion systems were the dominant types associated with biofilm formation in both *Leptolyngbyaceae* sp. and *Hyphomonadaceae* sp. However, type IX and VI were the major secretion systems related to biofilm formation in *Cytophagales* sp. (Fig. 4D). Thus, strong metabolic potentials related to EPS secretion and biofilm formation were observed in the core microbiota (*e.g. Leptolyngbyaceae* sp., *Cytophagales* sp.) of the BAC.

### Metabolic potentials related to the biofilm formation in MAGs

**Fig. 5:**
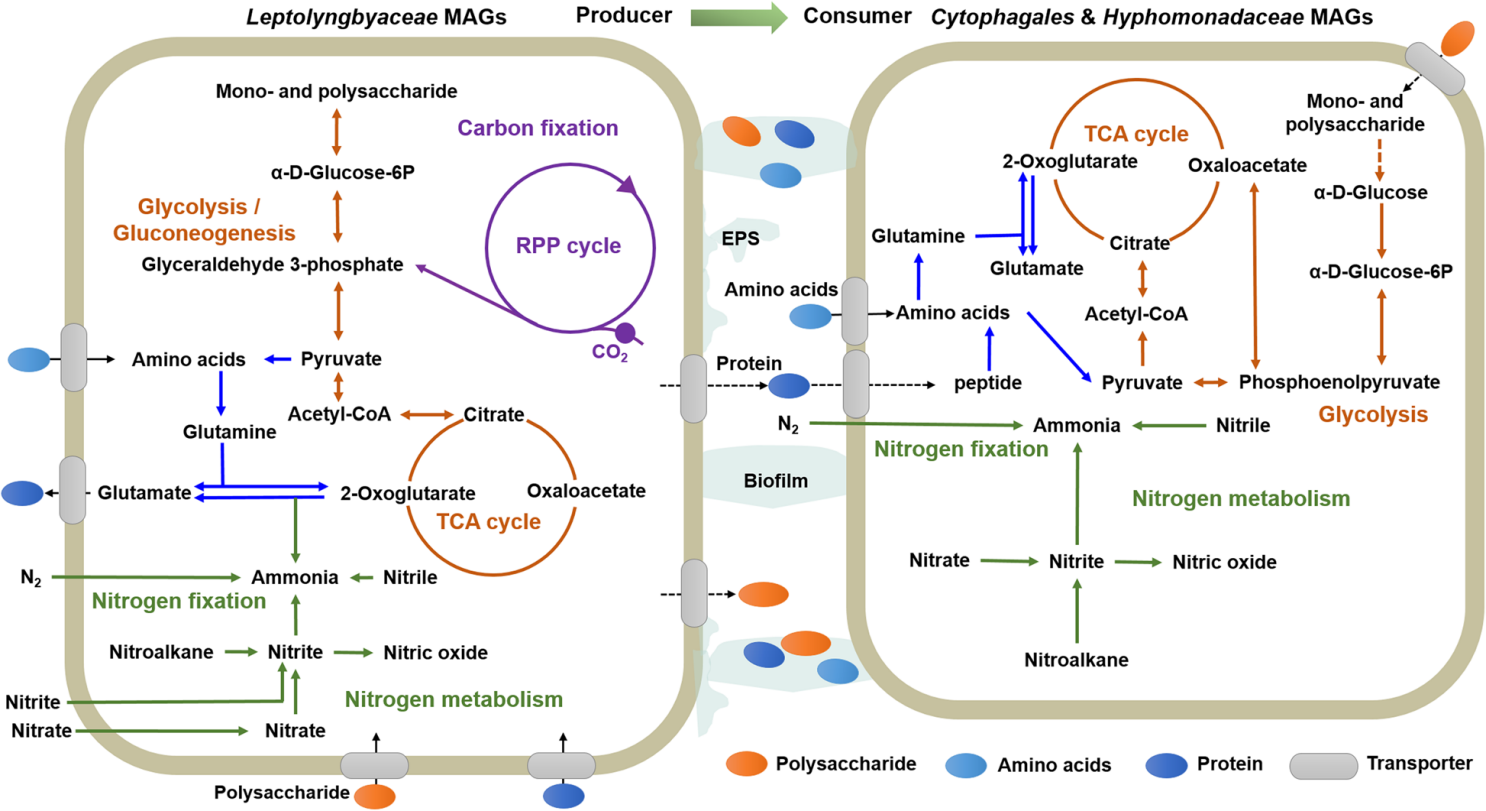
Overview of metabolic potentials in represented metagenome-assembled genomes (MAGs) of biological aqua crust (BAC). Genes related to polysaccharide, amino acid, and protein metabolism; RPP (reductive pentose phosphate) cycle; TCA cycle; and glycolysis or gluconeogenesis pathways are shown. The figure was created using Microsoft PowerPoint (2019) software. Detailed information of genes is provided in Table S9

The metabolic pathways related to carbon and nitrogen metabolism were reconstructed to further reveal the functional traits of phototrophic/diazotrophic biofilm formation with representative MAGs, *i.e. Leptolyngbyaceae* sp. (bins.302, bins.349), *Cytophagales* sp. (bins.15), and *Hyphomonadaceae* sp. (bins.64) (Fig. 5, Table S9). The reductive pentose phosphate cycles (RPP cycle) is the major process for carbon fixation, while nitrate reduction is the primary pathway for nitrogen metabolism in photoautotrophic *Leptolyngbyaceae* sp. Glyceraldehyde 3-phosphate (G3P) is the key intermediate linking the RPP cycle and glycolysis/gluconeogenesis pathways in *Leptolyngbyaceae* sp. Notably, complete pathways for nitrogen fixation, glycolysis/gluconeogenesis, and TCA (citric acid) cycles were present in the genomes of both *Leptolyngbyaceae* sp. and *Hyphomonadaceae* sp., suggesting that these highly abundant diazotrophs provided substantial nitrogen input to the BAC system. Interestingly, amino acids (*e.g.* glutamate), extracellular proteins, monoaccharide, and polysaccharides secreted by photoautotrophs (*e.g. Leptolyngbyaceae* sp.) are not only important carbon sources for heterotrophs (*e.g. Cytophagales* sp. and *Hyphomonadaceae* sp.) but also contribute to the EPS accumulation in photoautotrophic biofilm.

## Discussion

Biological aqua crusts (BACs) generally survive well in harsh ecosystems containing low levels of nutrients but high concentrations of heavy metals and metalloids [1]. Such features of BAC may strongly influence the microbial community diversity, particularly with low energy input during mining activities [18]. With this concern, efficient methods for metal(loid) removal in these harsh ecosystems, together with the underlying mechanisms at the genomic level for bioremediation, have rarely been reported in the literature [12]. In this study, we demonstrated that many biofilms, biofilm-forming microorganisms, and functional genes related to biofilm formation and metal(loid) bioremediation are distributed in the BAC. Considering the significant filtration of metal(loid)s and nutrients, we found that photoautotrophic and diazotrophic microorganisms could not only promote biofilm formation, but also contribute to metal(loid) bioremediation with great potential.

### Biofilm drove the enhancement of metal(loid) remediation

In stream ecosystems, biofilms composed of EPS (dry mass > 90%) and microorganisms (dry mass < 10%) have been depicted as ecosystem engineers, which can not only bind fine particles together but also accumulate a certain amount of nutrients such as carbon and nitrogen [12, 19]. In this study, both biogenic elements (*e.g.* carbon and nitrogen) and toxic metal(loid)s (*e.g.* Cd and As) were shown to be highly enriched in the BAC. Nutrients and metal(loid)s can accumulate from phototrophic and diazotrophic microorganisms in the form of biofilms in the BAC system. Several physicochemical and biological processes that drive the enhancement of the metal(loid) remediation may be involved in the absence or presence of biofilms. First, the introduction of metal(loid) ions (*e.g.* Pb and Cd) and mineral particles (*e.g.* clay minerals and metal oxides) with mine drainage results in the secretion of abundant and diverse EPS, which could contribute to metal(loid) immobilization during biofilm formation [14, 20, 21]. Second, many mineral particles (*e.g.* clay minerals and Fe/Mn oxides), which performed well in metal(loid) immobilization, are aggregated in the processes of biomineralization, coprecipitation and/or biosorption by the glue-like EPS of biofilms in aquatic ecosystems [11, 22]. Finally, the capacity of metal(loid) remediation could further be enhanced by mineral-based microaggregates that are comprised of active organic matter (*i.e.* microbial EPS) and functional minerals. Thus, metal(loid) availability and biofilm formation may be the two key biological traits in the BAC, whereas metal(loid)s and mineral particles aggregated from water bodies could drive the enhancement of metal(loid) remediation by accelerating biofilm formation.

### Genomic underpinnings of phototrophic and diazotrophic biofilm formation

As “dark matter of biofilms”, microbial EPS with complex components (*e.g.* polysaccharides, proteins) is an important prerequisite for biofilm formation [23, 24]. In this study, microorganisms, especially photoautotrophs and diazotrophs, were identified to have a strong potential for EPS secretion and biofilm formation, based on the metabolic characteristics revealed by metagenomics. Moreover, our study suggested that the accumulation of nutrients and metal(loid)s in BAC could be largely attributed to functional microorganisms, such as photoautotrophic *Leptolyngbyaceae* sp. and heterotrophic *Cytophagales* sp., which are related to EPS (*e.g.* extracellular peptidase, CAZymes) secretion and biofilm formation. Notably, the exopolysaccharide produced by microorganisms (*e.g. Pseudomonas aeruginosa*) is an important signal that stimulates biofilm formation [25], whereas the algae-derived CAZymes are the foremost means for heterotrophic bacteria to assimilate polysaccharides in epilithic biofilms of stream ecosystems [12]. In this study, the dominant taxa of *Leptolyngbyaceae* sp. and *Cytophagales* sp. showed strong secretion capacity for extracellular peptidase (*e.g.* families S9, S1, and S8) and CAZymes (*e.g.* families CBM50 and GT2). Moreover, type IX secretion system (T9SS), which are only found in the *Bacteroidetes*, could energize protein secretion and gliding motility through its specific components (*e.g.* extracellular enzymes and adhesins) [26, 27]. Gliding motility is an important characteristic of biofilm colonization and formation [28]. Given that the T9SS involves metal ion (*e.g.* Ca^2+^, Mg^2+^) assimilation, cellulose degradation, and biofilm formation by protein (*e.g.* adhesins) secretion [29, 30], our findings suggest that the most dominant taxa, *Cytophagales* sp., with T9SS as the dominant secretion type in the BAC system, may play an important role in regulating the geochemical behaviors of metal(loid)s and contributing to the biofilm formation.

In addition, genes related to biofilm formation in the dominant taxa (*e.g. Cytophagales* sp. and *Leptolyngbyaceae* sp.) of the BAC system were mainly represented by *OmpR*, *CRP* and *LuxS*. Gene *OmpR* can strongly enhance the initial adhesion capacity of microorganisms on abiotic surfaces to promote biofilm formation [31], whereas *CRP* is an important bacterial biofilm activator that is related to exopolysaccharide biosynthesis and transportation [32]. Protein LuxS encoded by gene *LuxS* is a ‘universal’ signal molecule of quorum sensing for the production of autoinducer AI-2 by microorganisms (*e.g. Vibrio harveyi* and *Escherichia coli*) [33–35]. Based on the genomic evidence of CAZymes, extracellular peptidase, secretion type (*e.g.* T9SS), and biofilm formation (*e.g.* quorum sensing), we propose that the dominant taxa (*e.g. Leptolyngbyaceae* sp. and *Cytophagales* sp.) with strong secretion capacity may contribute to biofilm formation and metal(loid) bioremediation in the BAC of polymetallic mine drainage.

### Ecological paradigm of metal(loid) remediation in oligotrophic environments

In mining ecosystems, autotrophic strategies, including carbon and nitrogen fixation, are often used by functional microorganisms in response to nutrient deficiency and metal(loid) (*e.g.* Cd and As) stress [36–38]. In this study, key functions, such as EPS secretion (*e.g.* CAZymes, extracellular peptidase) and biofilm formation were observed in the dominant taxa represented by *Leptolyngbyaceae*, *Cytophagales*, and *Rhodobacteraceae*. In line with the finding that *Rhodobacteraceae* are the only key microbiota in seawater biofilm [39], this study suggests that the dominant taxa (*i.e. Leptolyngbyaceae* and *Cytophagales*) in BAC is the newly discovered core microbiota in the initial biofilm of the water ecosystem. Biofilms are usually known as important contributors to environmental remediation, such as metal ion adsorption and biomineralization [11, 13]. Owing to their viscosity traits, microbial EPS can bind the fine particles together and stabilize the aggregates in the form of biofilms, which are critical for unstable and nutrient-limited river ecosystems [19, 40]. Moreover, strong abilities related to quorum sensing (*e.g.* gene *luxs*) and EPS secretion (*e.g.* CAZymes) were found in microorganisms classified as *Leptolyngbyaceae* and *Cytophagales*, suggesting that the biological processes associated with biofilm formation were widespread in the studied BAC system (Fig. 2). Furthermore, our previous study also showed that mineral particles (*e.g.* clay minerals and Fe/Mn oxides) are cemented by the filamentous *Cyanobacteria* and EPS in the form of aggregates in the BAC of mine drainage [1]. Therefore, we assume that microbial functions related to biofilm formation are not only ubiquitous in the BAC system but are also critical for microbial adaptation to the oligotrophic habitats studied here.

**Fig. 6:**
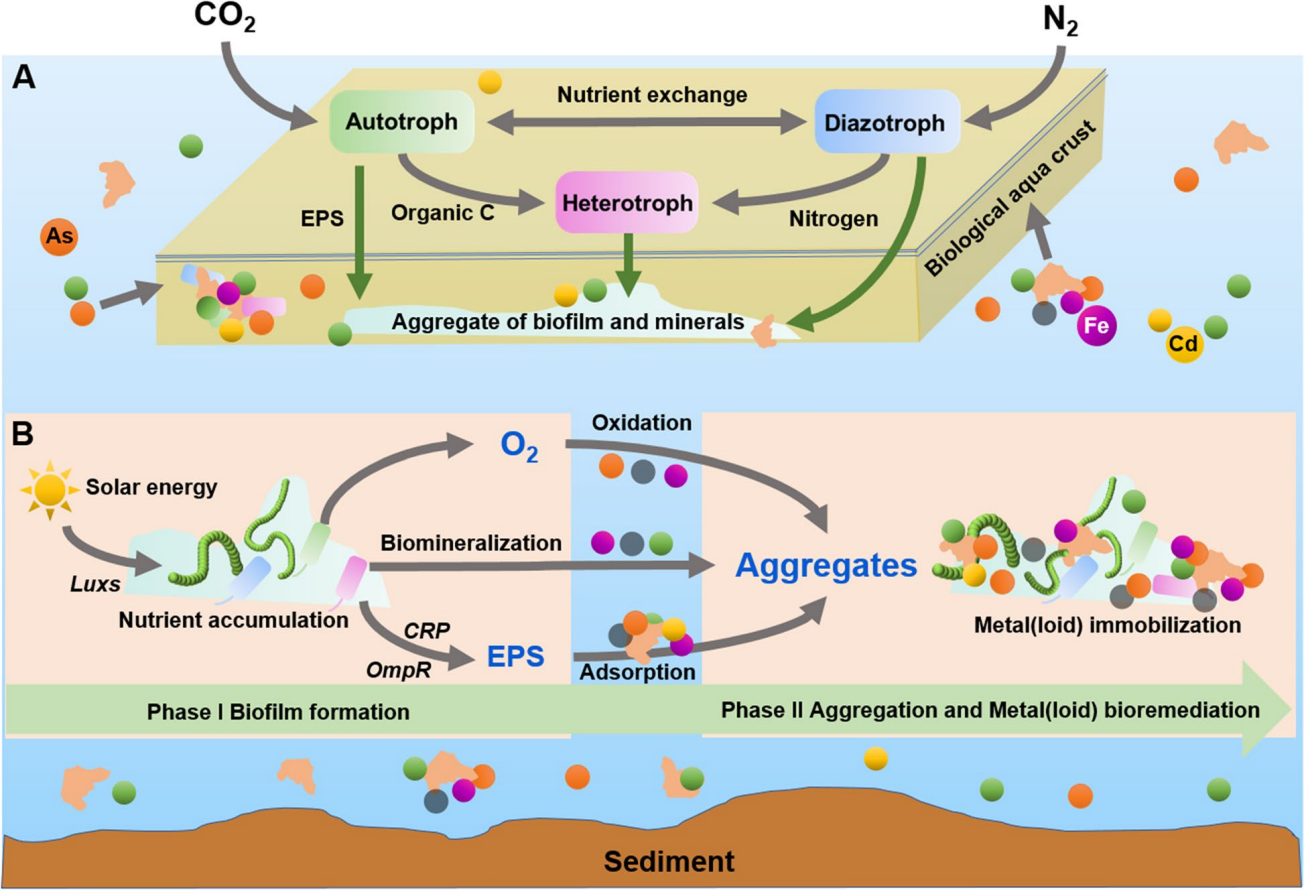
Conceptual diagram showing the potential interaction on nutrient exchange (**A**) and metal(loid) bioremediation (**B**) driven by biofilm-forming microorganisms in biological aqua crust of oligotrophic environments. Microsoft PowerPoint (2019) software was used for the diagram creation

In addition, photoautotrophs (*e.g. Cyanobacteria*) are important producers (Fig. 5) in the ‘green’ food web in the epilithic biofilms of oligotrophic streams [12]. As the genetic shortcomings of nitrogen fixation occurred in photoautotrophic pioneer (*e.g. Microcoleus vaginatus*) of biological soil crusts, symbiotic nutrient (*e.g.* C, N) exchange between photoautotrophs and heterotrophic diazotrophs is an important adaptive strategy for cyanosphere microorganisms in extreme desert environments [41, 42]. This study also found a potential linkage between photoautotrophs and heterotrophs in nutrient exchange by reconstructing the metabolic pathways of the dominant taxa (*e.g. Leptolyngbyaceae* sp., *Cytophagales* sp.) in the BAC system (Fig. 6A). As one of the dominant taxa in the BAC, *Leptolyngbyaceae* functioning as photoautotrophs and diazotrophs might not only occupy crucial ecological niches in the BAC but also govern the geochemical behaviors of inorganic particles that are rich in metal(loid)s in these biological materials of aquatic ecosystems owing to their powerful capacity for filamentous winding and biofilm formation.

Although inorganic particles have been reported to weaken the structural stability of biofilms [43], our work suggests that minerals and metal(loid)s together with EPS would greatly enhance their mechanical stability, as well as the potential for *in situ* bioremediation of metal(loid)-contaminated waterbodies. Here, we propose a conceptual model to illustrate the important role of dominant taxa, especially autotrophic *Cyanobacteria* in metal(loid) bioremediation using solar energy in oligotrophic stream ecosystems (Fig. 6B). First (Phase I), photoautotrophs, especially *Leptolyngbyaceae* sp., could not only absorb plenty of solar energy in sunshine-abundant environments but also proliferate through quorum sensing (*e.g.* gene *luxS*) and then store the energy in the form of a biofilm. Nutrient (*e.g.* C and N) accumulation mediated by *Leptolyngbyaceae* sp. and *Hyphomonadaceae* sp. was also completed during the process of biofilm formation. Furthermore (Phase II), filamentous microorganisms, such as *Leptolyngbyaceae* sp. could physically bind to or adsorb fine mineral particles (*e.g.* clay minerals and Fe/Mn oxides) from polymetallic mine drainage. Notably, the viscous EPS including exopolysaccharides and exoproteins, which are produced by the biofilm-forming microorganisms (*e.g. Leptolyngbyaceae* sp., *Cytophagales* sp.), could act as “glue” for fine particles and then promote the formation of organo-mineral aggregates with stable structures [19, 44]. Finally, the autotrophic biofilm-mineral aggregates represented as porous BAC could strongly contribute to metal(loid) (*e.g.* Pb, As and Cd) bioremediation by oxidation, biosorption and coprecipitation in the current and previous studies [1]. Thus, large amounts of metal(loid)s can be simultaneously immobilized from mine drainage during BAC formation.

## Conclusions

Our genome-resolved metagenomic analyses revealed the mechanism by which the dominant microbiota in BAC allow phototrophic/diazotrophic biofilms to contribute to the metal(loid) bioremediation in oligotrophic environments. Taken together, our findings provide a novel perspective on future metal(loid) bioremediation with nature-based solutions; that is, phototrophic/diazotrophic biofilm could control metal(loid) bioremediation using solar energy in oligotrophic and harsh ecosystems, such as mine drainage. Further studies are needed to substantiate our observations regarding the formation mechanisms driven by the dominant microbiota in the BAC of aquatic ecosystems.

## Materials and methods

### Site description and sample collection

The study site was selected in an abandoned Pb/Zn tailing wetland (24.382116° N, 116.213768° E) in Meizhou, Guangdong Province, China, which has been detailed in previous studies [1, 45, 46]. Samples of biological aqua crust (BAC) and source tailings together with unstable physical crust were collected from a tailing wetland [1]. Five replicate samples were collected from three sites, *i.e.* source tailings (control, C_1–5_, B_0_ group), upstream BAC (UB_1–5_, B_1_ group) and downstream BAC (DB_1-5_, B_2_ group) (Table S2). Samples were collected in Petri dishes (*ϕ* 9 cm, Corning, USA) for geochemical analysis and characterization and in sterile centrifuge tubes (50 mL, Corning, USA) for microbial analysis. Samples for molecular analysis were immediately transported at -18 °C to the laboratory, and then were stored at -80 °C until further processing.

### Geochemical analyses and morphology characterization

Samples in dishes were dried using a freeze-dryer (Labconco, USA), thoroughly ground, and sieved (100-mesh) before geochemical analysis. The measurement details of the total organic carbon (TOC) and microstructure characterization (*i.e.* stereomicroscopy, FESEM and TEM) are described in a previous study [1]. Briefly, total organic carbon (TOC) was measured using the wet oxidation (5 mL K_2_Cr_2_O_7_, 7.5 mL H_2_SO_4_) method [47]. SEM analysis was conducted using freeze-dried samples coated with carbon, whereas TEM analysis was performed using the cross-sectional samples embedded in epoxy resin. Total nitrogen (TN) was determined using an elemental analyzer (Vario EL Cube, Elementar, Germany). The potentially bioavailable fractions of metal(loid)s in the samples were extracted using 1.0 mol L^−1^ HNO_3_ [48]. After digestion using *aqua regia* (HCl: HNO_3_ = 3: 1), total phosphorus (TP) and potentially bioavailable fractions of the metal(loid)s were determined by inductively coupled plasma optical emission spectrometry (ICP-OES) (Optima 5300DV, Perkin-Elmer, USA). Standard reference materials and reagent blanks were used to ensure analytical accuracy.

### DNA extraction, 16S rRNA gene amplicon sequencing, and bioinformatic analyses

Total genomic DNA was extracted from each freeze-dried sample using the FastDNA® SPIN Kit for Soil (MP Biomedicals, France) following the manufacturer’s protocol. DNA quality and concentration were assessed by spectrometry absorbance using a NanoDrop 2000 spectrophotometer (Thermo Fisher Scientific, USA). The V4 hypervariable region of 16S rRNA gene was amplified with primers 515F (5′-GTGYCAGCMGCCGCGGTAA-3′) and 806R (5'-GGACTACHVGGGTWTCTAAT-3'). High-throughput sequencing of 16S rRNA genes was conducted by Biomarker Technologies Corporation (Beijing, China) using the Illumina Novaseq 6000 platform [49]. The obtained sequences with paired-end reads were then overlapped, merged, and preprocessed using the QIIME2 package (2020.8) [50]. Briefly, raw sequences were denoised using the DADA2 pipeline, and sequences containing ambiguous bases, chloroplast, mitochondria, chimeras, and low-abundance sequences (quality score < 20) were removed [51]. The obtained amplicon sequence variant (ASV) table summarized by the representative sequences was used for downstream analyses. The normalized sequences were taxonomically assigned with the SILVA 138 database [52, 53].

### Metagenomic and binning analyses

Based on amplicon analysis, six BACs from B_1_ and B_2_ were selected for metagenomic analysis to investigate the metabolic potentials of the microbiota (Table S2). Metagenomic libraries were constructed and sequenced using an Illumina NovaSeq 6000 platform (Table S3). A total of 143,183,979,090 raw reads were generated from the six metagenomes. The paired-end raw data were filtered using Trimmomatic (v 0.36) tool [54], and 381,352,622 high-quality clean tags were obtained. The clean reads were then de novo assembled using MEGAHIT (v 1.1.2) software to trim any contigs shorter than 300 bp. QUAST (v 2.3) software was then employed for quality assessment of the metagenome assemblies, and 7,928,870 assembled contigs were obtained [55]. MetaGeneMark (v 3.26) software was used for gene prediction with default parameters, and 11,340,914 genes were identified [56]. The cd-hit (v 4.6.6) software was used to build a non-redundant gene set (5,834,387 genes) with thresholds of 95% similarity and 90% coverage [57].

In addition, the metabat2 (v 2.12) software was employed for contig binning with ≥ 90% bin completeness and ≤ 5% redundancy to clarify the metabolic potentials of microbiota in the BAC [58]. After being assessed by CheckM (v 1.0.6) software, a total of 109 metagenome-assembled genomes (MAGs) with high quality were obtained for detailed downstream analysis (Table S4). The relative abundance of MAGs was quantified using BWA software [59]. The phylogenomic tree was constructed with reference to the method described in previous study [60]. Phylogenetic analyses of the concatenated ribosomal protein genes were performed using Anvi’o (v 6.1) software [61] based on the hidden Markov Model profile [62]. The best-fit model and 1000 ultrafast bootstrap tests were implemented using the IQ-TREE (v 1.6.12) software [63] to construct maximum-likelihood trees. Phylogenetic trees constructed based on these MAGs were visualized using iTOL tool [64]. The taxonomic annotation of MAGs was blasted against the GTDB database [65]. Prodigal software (v 2.6.3) was used to predict the protein-coding regions [66]. The protein-coding regions were annotated using a BLAST search against the NCBI nr database, KEGG server (BlastKOALA) [67], eggNOG-mapper (v 5.0.0) [68], and InterProScan tool (v 5.44–79.0) [69] to reconstruct the metabolic pathways of selected MAGs in the BAC. The R (v 4.0.3) packages (*e.g.* “ggplot2” and “reshape2”) were used for data visualization [70, 71].

## Supplementary Information

**Additional file 1: Fig. S1.** Microbial composition of amplicon sequence variants (ASVs) at the phylum level in studied samples. Biological replicates (*n* = 5) are showed in separate stacked bars. B_0_ represent the source tailings, and B_1-2_ stands for the BACs collected at Sites 1 and 2.

**Additional file 2: Table S1.** The mean relative abundances of the high-abundant microbiota in the biological aqua crust and source tailings. **Table S2.** Summary of the sample names for amplicon and metagenome analysis. **Table S3.** Summary of the quality controlled and assemblage metagenomic data in studied BAC. **Table S4.** Summary of the MAGs information in studied biological aqua crust. **Table S5.** Summary of the biofilm genes in MAGs of studied biological aqua crust. **Table S6.** Genes involving in CAZyme secretion of the biological aqua crust. **Table S7.** Genes encoding extracellular peptidase in selected MAGs of the biological aqua crust. **Table S8.** Genes related to the secretion type of biological aqua crust. **Table S9.** Genes related to the metabolic pathways of biological aqua crust.

## Data Availability

Raw 16S rRNA amplicon sequencing datasets are available in the GSA database under the project PRJCA010655 (CRA007730). The newly constructed MAGs have been submitted to the NCBI database under the project PRJNA866711 with the accession numbers from SAMN30172104 to SAMN30172212. The R code used in this study is available upon reasonable request.
