# Supplementary material for: Novel biological aqua crust enhances in situ metal(loid) bioremediation driven by phototrophic/diazotrophic biofilm
Source: Microbiome. 2023 May 18;11:110. doi: 10.1186/s40168-023-01549-3 (PMC10193787; doi:10.1186/s40168-023-01549-3)
Supplement: Supplementary file 2 — Additional file 1: Fig. S1. Microbial composition of amplicon sequence variants (ASVs) at the phylum level in studied samples. Biological replicates (n = 5) are showed in separate stacked bars. B0 represent the source tailings, and B1-2 stands for the BACs collected at Sites 1 and 2. [file 40168_2023_1549_MOESM1_ESM.docx]

**Novel biological aqua crust enhances *in situ* metal(loid) bioremediation driven by phototrophic/diazotrophic biofilm**

Guobao Wang^1, 2^, Xiuran Yin^3^, Zekai Feng^1^, Chiyu Chen^1^, Daijie Chen^1^, Bo Wu^1, 2^, Chong Liu^4^, Jean Louis Morel^5^, Yuanyuan Jiang^1^, Hang Yu^1^, Huan He^6^, Yuanqing Chao^1, 2^,
Yetao Tang^1, 2^, Rongliang Qiu^1, 2, 6^**^*^**, and Shizhong Wang^1, 2^**^*^**

^1^ School of Environmental Science and Engineering, Sun Yat-sen University, Guangzhou, 510006, China

^2^ Guangdong Provincial Key Laboratory of Environmental Pollution Control and Remediation Technology, Sun Yat-sen University, Guangzhou, 510275, China

^3^ Microbial Ecophysiology Group, University of Bremen, Bremen, Germany

^4^ Institute of Agricultural Resources and Environment, Guangdong Academy of Agricultural Sciences, Guangzhou 510640, China

^5^ Laboratoire Sols et Environnement, UMR 1120, Université de Lorraine, INRAE, 54518 Vandoeuvre-lès-Nancy, France

^6^ Guangdong Laboratory for Lingnan Modern Agriculture, Guangdong Provincial Key Laboratory of Agricultural & Rural Pollution Abatement and Environmental Safety, College of Natural Resources and Environment, South China Agricultural University, Guangzhou 510642, China

*Corresponding author(s): Shizhong Wang ([wshizh2@mail.sysu.edu.cn](mailto:wshizh2@mail.sysu.edu.cn)), Rongliang Qiu ([eesqrl@mail.sysu.edu.cn](mailto:eesqrl@mail.sysu.edu.cn)).

**Supplementary figures**


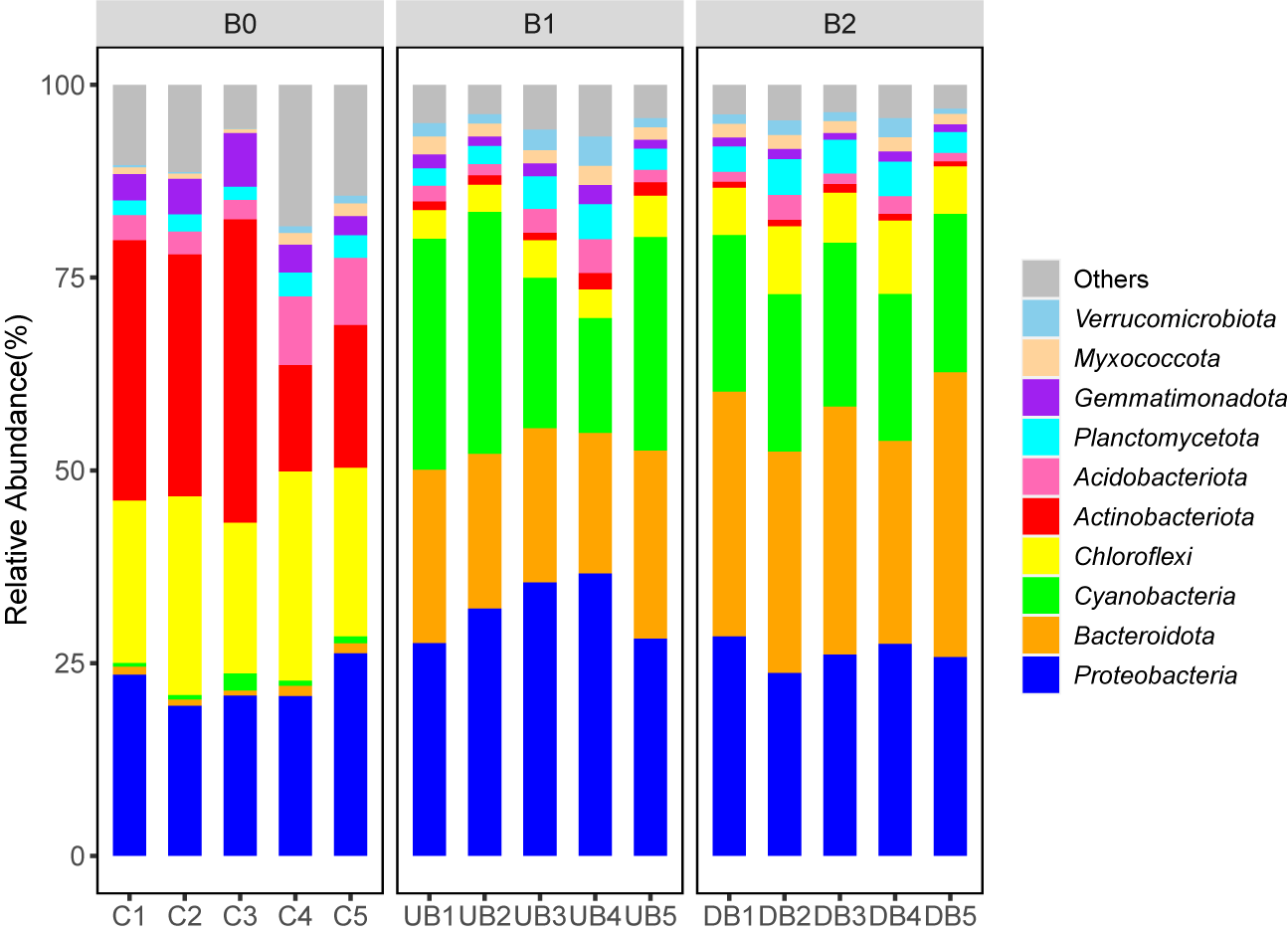


**Fig. S1** Microbial composition of amplicon sequence variants (ASVs) at the phylum level in studied samples. Biological replicates (n = 5) are showed in separate stacked bars. B_0_ represent the source tailings, and B_1-2_ stands for the BACs collected at Sites 1 and 2.
